# The Coronavirus Pandemic as a Game-Changer: When NBA Players Forced America to Think. Again.

**DOI:** 10.3389/fpsyg.2020.600267

**Published:** 2021-01-05

**Authors:** Yair Galily

**Affiliations:** Interdisciplinary Center Herzliya, Herzliya, Israel

**Keywords:** NBA, #BlackLivesMatter, LeBron James, activisim, COVID - 19

*Alone we can do so little; together we can do so much*.

Helen Keller

On August 26, 2020, some of the best-known athletes in the world decided to act. Players from the National Basketball Association's Milwaukee Bucks did not arrive for their playoff game against the Orlando Magic and the league was forced to cancel the match. The Bucks refused to play the fifth game of the series in protest against the firing of an unarmed black man, Jacob Blake, in Kenosha, Wisconsin, only few miles from where they usually play. Many teams in other American leagues followed the Bucks and Magic. Players from all NBA teams held emergency meetings, during which players requested to end the season immediately.

Another major turning point related to the NBA occurred 5 months earlier, on March 18, 2020. Rudy Gobert from the Utah Jazz was the first player in the league to test positive for COVID-19 and the governors of the states decided it was time to bring many daily activities to a halt. The event that made Americans realize that COVID-19 was not going to disappear within a week or two was not President Donald Trump's announcement of a partial closure of the skies for flights from Europe that day, but rather a decision by NBA Commissioner Adam Silver to stop the NBA season. Some countries returned to their routines faster than others (and their economies paid a price for it), but 2020 will probably be remembered as a year in which the pandemic was a real turning point.

Silver came up with a plan for how the season could still be saved during the summer, but a lot has happened in the intervening months. The killing of African-American man George Floyd by a white policeman, the Black Lives Matter protests, and Florida becoming a COVID hotspot, were not in the commissioner's plans when he announced that Orlando would host all league teams in a bubble that would allow the season to end at the end of July; therefore, the NBA was back playing in sub-optimal conditions.

The NBA administration worked not only to keep players healthy inside the bubble, but allowed players to express themselves to an unprecedented degree. The players recognized the attention that was on them and saw the tournament as an opportunity to convey messages that would run much deeper than just basketball. The impact of the struggle for black rights in America following Floyd's death has been largely due to the league's current and past stars. When LeBron James, Steph Curry, and Giannis Antetokounmpo joined the protest and sometimes even marched alongside the protesters, the world listened. Stephen Jackson, a close friend of Floyd who had played basketball at Golden State and Indiana, has become one of the symbols of the struggle.

Sport-based activism refers to specific actions taken by athletes to alter and mitigate the hegemonic nature of structural arrangements, rules/policies/bylaws, and practices through sport organizations that serve to reinforce subordination, marginalization, and exploitation of certain groups (Cooper et al., 2019, p. 22). For many years, American athletes have spoken out about social and political issues; examples include Muhammad Ali, Jackie Robinson, Bill Russel, Tommie Smith (with John Carlos), Mahmoud Abdul-Rauf, Craig Hodges, Billie-Jean King, and, more recently, Colin Kaepernick. Most of these athletes paid a price, in terms of financial penalties caused by speaking out on polarizing topics, that current athletes, especially minorities, are less likely to pay (Coombs and Cassilo, 2017).

As many sport scholars, including myself, have claimed, sport does not operate in isolation from broader society. Instead, sport serves as a site where societal inequalities such as racism, sexism, economic stratification, and other forms of oppression are reproduced, exacerbated, and/or ignored (Cooper et al., 2019; Galily, 2019).

However, sport, like other cultural domains, is torn between spirit and matter. On one hand, sport is primarily about values—the personal stories of the competitors, striving for excellence, commitment to the team, and adherence to a goal. On the other hand, the laws of business stipulate that the show must go on for the hundreds of millions of viewers around the world. The NBA has a turnover of over USD$10 billion a season, and such a business has little room for mistakes or changes.

While the American basketball realm includes many stakeholders—the owners, the advertising and sales people, the field workers, and the media—the league ultimately belongs to the players. Fans look up to them and their special status gives them tremendous power to influence and change reality.

On October 4, 2019, at the height of protests in Hong Kong against the allegedly repressive Chinese regime, Daryl Murray, the general manager of the Houston Rockets, tweeted an expression of support for the protesters: “Fight for freedom. Stand with Hong Kong.” The tweet caused a stir among Chinese spectators, who make up about 10–15 percent of all NBA fans, and many called for a boycott of league games. Chinese broadcasters announced they would stop broadcasting the games. The league, fearing huge losses worth billions of dollars due to the loss of Chinese crowds, was quick to rectify the situation. Murray deleted the tweet and apologized, and even superstar player LeBron James was recruited to express a position that he does not support Murray's position and that he does not oppose Chinese regime action. This point event demonstrated that the red line passes where revenue begins to hit.

However, it appears that when it comes to America—and, this time, when it comes to the African-American community in the US—the players have chosen not to remain silent and to stand *together*. Regardless of the economic consequences, NBA players decided to boycott the playoffs as a protest against US police violence. Players listen to their audience and public mood, and have used their status as cultural heroes and influence on social media to advance the values and goals of the #BlackLivesMatter protest. The latest incident has changed the future rules of the game between the players, who are trapped in fat but binding contracts, and the giant corporations of the teams in the league when it comes to the right to protest. As I have argued elsewhere (Galily, 2019), by mastering social media in general and Twitter in particular, NBA players today are reaching far more people who, under other circumstances, would not pay attention to the political issues that athletes are discussing (Xu and Zhou, 2020).

## A (Real) Game Changer

On March 2012 before a game against Detroit, LeBron James recruited Miami players to protest by wearing hooded sweatshirts after the shooting of Trevion Martin. This started a new wave of action among the athletes who held the power and money the system depends on. The years that followed were marked by incidents such as the strangulation of George Floyd by police in New York, which led to athletes wearing “I Can't Breathe” shirts; or five St. Louis Rams' players entering game with their hands raised as a tribute to the “Hands Up, Don't Shoot” protest after Michael Brown was shot by police in Ferguson, Missouri; and certainly Colin Kaepernick, who knelt during the national anthem before a game in 2016, which became a picture of protest and a central and divisive discourse in America. After decades of coming to terms with the current situation, the previous decade was accompanied by dozens of athletes taking an active part in protests against what they felt was built-in discrimination in America, and especially police violence (Mudrick et al., 2019).

Regardless of whether the more recent incidents are a continuation of the same wave or a new wave, the system is changing. Everyday life has been turned upside to an almost unprecedented degree during COVID-19, so the current protests that also unite other pressure groups besides the African-American population have led to a change in public opinion, even among the wealthy and decision-makers. The same NFL that condemned Kaepernick moved to support protesting athletes. The US Olympic Committee that dumped Smith and Carlos after the 1968 Mexico City Olympics' power salute went on to support the right of athletes to protest. The US Soccer Association that banned Megan Rapinoe for kneeling during the national anthem (one of the first white athletes to actively join Kaepernick's protests) also eventually moved to support the protests. Alongside established sportspeople, there have also been new voices of athletes, such as the abovementioned Stephen Jackson, 16-year-old Coco Gauff who speaks in exceptional adulthood, or Bubba Wallace, the only African-American NASCAR driver. Wallace himself did not define himself as a social activist until the last wave of protests, when he began to learn more and worked for protests in NASCAR races and the removal of Confederate flags from races in one of the industries that is most closely identified with Southern, white, Republican audiences.

The protests had an international impact. In the United Kingdom, for example, Arsenal and Manchester City players knelt before an English Premier League game, Marcus Rashford took aim at child poverty with a new taskforce, and even the International Olympic Committee allowed the Athletes Committee to recommend how to deal with protests consistent with the Olympic spirit. Whether because of the pandemic or because of the protests, a new social order is being formed.

At the same time, however, it might worth mentioning, as basketball star Kareem Abdul-Jabbar did, that “It's great that athletes are speaking out. But some of them are spouting nonsense.” He was referring to American Volleyballer Kerri Jennings, who posted on Instagram an extended rationalization for going shopping without a mask, which she called “a little exercise in being brave” and faced a lot of criticism. Another example is tennis star Novak Djokovic, who not only organized a tournament at the peak of the pandemic and contracted COVID-19, alongside his wife and many who participate without masks or social distancing, but subsequently stated that he would have done it again since he did not break any law. Abdul Jabbar commented:

“… Being responsible role models during a worldwide pandemic that has killed nearly a million people isn't about laws; it's about concern and respect for the lives of others, especially the others who can't afford to miss work or don't have access to good healthcare.” (Abdul-Jabbar, 2020).

As Sunkara (2020) correctly observed recently, NBA players might not be common laborers, but they have far more in common with the working-class communities that supported so many of them in youthful than with the moguls they now work for. The COVID-19 pandemic era has been an opportunity to make a difference, and just like all workers along history, they have to consolidate collectively. As journalist Branko Marcetic put it: “Millions of people are seeing workers they admire, even idolize, effectively striking in solidarity with a bigger cause” (Marcetic, 2020). As sports can provide a guide for young people (and athletes) to learn concepts of teamwork, leadership, work ethic and integrity, we ought to add social justice.

## Author Contributions

The author confirms being the sole contributor of this work and has approved it for publication.

## Conflict of Interest

The author declares that the research was conducted in the absence of any commercial or financial relationships that could be construed as a potential conflict of interest.
